# Influence of Maternal Infection and Pregnancy Complications on Cord Blood Telomere Length

**DOI:** 10.1155/2021/3339456

**Published:** 2021-09-25

**Authors:** Keith T. S. Tung, Catherine M. W. Hung, Ko Ling Chan, Rosa S. Wong, Hing Wai Tsang, Wilfred H. S. Wong, Camilla K. M. Lo, Winnie W. Y. Tso, Gilbert T. Chua, Benjamin K. Yee, Ian C. K. Wong, W. C. Leung, Patrick Ip

**Affiliations:** ^1^Department of Paediatrics and Adolescent Medicine, The University of Hong Kong, Hong Kong SAR, China; ^2^Department of Obstetrics and Gynaecology, Kwong Wah Hospital, Hong Kong SAR, China; ^3^Department of Applied Social Sciences, The Hong Kong Polytechnic University, Hong Kong SAR, China; ^4^Department of Rehabilitation Sciences, The Hong Kong Polytechnic University, Hong Kong SAR, China; ^5^Centre for Safe Medication Practice and Research, Department of Pharmacology and Pharmacy, The University of Hong Kong, Hong Kong SAR, China; ^6^Research Department of Practice and Policy, UCL School of Pharmacy, University College London, UK

## Abstract

**Background:**

Exposure to suboptimal intrauterine environment might induce structural and functional changes that can affect neonatal health. Telomere length as an important indicator of cellular health has been associated with increased risk for disease development.

**Objectives:**

This study was aimed to examine the independent and combined effects of maternal, obstetric, and foetal factors on cord blood telomere length (TL).

**Methods:**

Pregnant women at the gestational age of 20^th^ to 24^th^ week who attended the antenatal clinic of a major local hospital in Hong Kong were recruited. Participants were asked to complete a questionnaire on demographics, health-related quality of life, and history of risk behaviors. Medical history including pregnancy complications and neonatal outcomes was obtained from electronic medical records of both mother and neonate. Umbilical cord blood was collected at delivery for TL determination.

**Results:**

A total of 753 pregnant women (average age: 32.18 ± 4.51 years) were recruited. The prevalence of maternal infection, anaemia, and hypertension during pregnancy was 30.8%, 30.0%, and 6.0%, respectively. The adjusted regression model displayed that maternal infection was negatively associated with cord blood TL (*β* = −0.18, *p* = 0.026). This association became even stronger in the presence of antenatal anaemia, hypertension, delivery complications, or neonatal jaundice (*β* = −0.25 to −0.45).

**Conclusions:**

This study consolidates evidence on the impact of adverse intrauterine environment at the cellular level. Maternal infection was significantly associated with shorter cord blood TL in a unique manner such that its presence may critically determine the susceptibility of telomere to other factors.

## 1. Introduction

Emerging evidence from epidemiological, clinical, and experimental studies suggests that early adverse experiences may cause long-term health problems and increase susceptibility to disease [1, 2]. Particularly, it has been suggested that the developmental origins of health and disease can be traced back to the intrauterine life. As developing foetuses are very sensitive to the quality of intrauterine environment, adverse exposures during this period of rapid cell replication and differentiation might cause permanent structural and functional impairments [3] which may increase the risk for health problems in later years [4]. While evidence of link between suboptimal intrauterine environment and adverse health outcomes is available, limited research has investigated the impact of suboptimal intrauterine environment at the cellular level.

Telomere dynamics has only recently been studied as a candidate mechanism underlying the health consequences of suboptimal intrauterine environment [5]. Telomeres, the nucleoprotein complexes with specialized repetitive sequence of -TTAGGG- located at the distal end of each chromosome, mainly protect the genomic integrity against replication flaw [6]. It also acts as a buffer against abnormal fusion and nucleolytic degradation and thus plays a critical role in maintaining chromosomal structures during mitotic cell division. The length of telomere has been regarded as the life-clock of a cell regulating its replicative senescence and survival [7]. Telomere length (TL) varies substantially between individuals, as the rate of telomere shortening depends on factors such as age, gender, and environmental exposures [8]. Accelerated telomere shortening could potentially increase the risk for age-related diseases such as hypertension, cardiometabolic disorders, and immune-related diseases [9–11]. In addition, potential linkages between short telomeres and biological and psychosocial problems have been documented [10].

Numerous studies have examined the environmental and genetic determinants of TL and/or the rate of change in TL. Previous research on neonates has demonstrated associations between TL and time-invariant factors such as sex and race, parental age at conception, and parental engagement in risk behaviors during pregnancy [12–14]. However, conflicting results have been displayed on the role of genetic factors in the individual's variation in the cord blood TL [15–17]. The apparent inconsistent emphasis on genetic factors suggests that other unknown factors responsible for the interindividual variation in neonatal TL may exist. Indeed, emerging evidence has indicated that TL is highly vulnerable to specific events and exposures during the prenatal and early postnatal periods which may set the course for disease or health risk later in life [18–21].

There have been conflicting results as to what factors in the intrauterine environment may predispose individuals to abnormal telomere dynamics [15]. It is known that foetal TL is particularly susceptible to suboptimal intrauterine environment [22, 23]. Several animal and human observational and experimental studies found that suboptimal intrauterine conditions, such as obstetric complications, maternal stress, and maternal obesity, were associated with shorter foetal TL [24, 25]. Telomeres may also be shorter in neonates who are smaller for their gestational age and born preterm [26, 27]. However, these previous studies mainly focused on the impact of individual factors but not the combined influence of these factors. Furthermore, much of the research on TL has been conducted in western populations. It is therefore important to assess risk factors for abnormal telomere dynamics among nonwestern populations to enhance our understanding of racial differences in TL and/or rate of change in TL under adverse conditions [13, 28]. Therefore, the present longitudinal study utilized data from a cohort of pregnant women in Hong Kong to explore the independent and combined effects of maternal, obstetric, and foetal factors on cord blood TL.

## 2. Methods

### 2.1. Study Participants

This study involved pregnant women recruited at the gestational age of 20^th^ to 24^th^ week from the antenatal clinic of a major public hospital, Kwong Waah Hospital, in Hong Kong. During the period of January to April 2017, a total of 753 pregnant women consented to participate in this study. Informed consent was obtained from mothers on questionnaire completion, cord blood collection, and retrieval of their medical records. The mothers were asked to complete a questionnaire on their demographics, health-related quality of life, and history of risk behaviors. The babies' umbilical cord blood was collected in the labour room or operation theatre immediately after delivery by trained midwives. Patient's particulars, pregnancy complications, birth outcomes, and neonatal conditions were collected from the patient's records through the electronic medical record system. The research protocol was approved by the Institutional Review Board of the Hospital Authority Kowloon West Cluster Research Ethics Committee (reference number: KW/FR-16-042(97-01) [1]).

### 2.2. Measures

#### 2.2.1. Cord Blood TL

Genomic DNA was isolated and extracted from the collected cord blood samples using the QIAamp DNA Mini Kit (Qiagen). Upon quality checking and quantification of the extracted DNA, they were handled in triplicate to determine the average value of TL by quantitative polymerase chain reaction according to procedures described in previous literature [6]. TL was then quantified as a relative ratio of the telomere repeat copy number (*T*) to single-copy gene 36B4 copy number (*S*) using the formula of *T*/*S* = 2^−ΔCt^, where ΔCt is the mean difference between the threshold cycle (Ct) value of the 36B4 gene and telomere repeats.

#### 2.2.2. Maternal Obstetric and Foetal Outcomes

The mother's records of pregnancy complications were retrieved from the Clinical Data Analysis and Reporting System (CDARS). The CDARS is a database developed by Hospital Authority to capture clinical data including outpatient attendances and inpatient admissions of all public hospitals in Hong Kong. The International Classification of Diseases, 10th revision (ICD-10) system for diagnosis [29] was adopted in the CDARS to code different disease outcomes. In this study, we used the ICD-10 codes to identify mothers with hypertensive disorder “O13.1-O13.9, O16.1-O16.9,” infection “A49.1, O98.8,” or anaemia “D64.9, O99.0” during pregnancy. The complications at delivery were also identified using the ICD-10 codes “P07.1” for low birthweight, “P59.9” for neonatal jaundice, and “O41.8, O68.1, O99.0” for unspecific complication.

#### 2.2.3. Maternal Mental Wellbeing

The mother's mental wellbeing was assessed using the 12-item Short-form Health Survey, which is a validated questionnaire designed for assessing quality of life across eight domains including physical functioning, role limitations due to physical health, bodily pain, general health, vitality, social functioning, role limitations due to emotional health, and mental health [30]. In this study, the mental component summary score was used to measure the mother's overall mental wellbeing with a higher score indicating better wellbeing.

#### 2.2.4. Maternal History of Risk Behaviors

The mother was asked with the question “how often had you engaged in risk behaviors including cigarette smoking, alcohol drinking, and drug use?” on a 4-point scale ranging from 1 = never to 4 = always. Maternal responses on history of risk behaviors were dichotomised (never vs. ever engaged) and treated as a categorical variable in the analyses.

#### 2.2.5. Maternal and Neonatal Characteristics

Maternal demographics including age, educational level, marital status, employment status, and family income were obtained from the mother's self-report questionnaire. Neonatal characteristics including gender and birthweight were obtained from the CDARS.

### 2.3. Data Analysis

Descriptive statistics were first computed to describe the maternal and neonatal characteristics including incidences of medical complications during pregnancy and at delivery. Data were presented as mean (standard deviation) for continuous variables and frequency (percentage) for categorical variable. Cord blood TL was compared between groups of different characteristics using independent *T*-tests.

To examine the effect of complications during pregnancy and at delivery on cord blood TL, four sets of regression models were separately built. First, univariate analyses were conducted to examine the crude associations between single or combined complications and cord blood TL. In the second set, we adjusted the models for maternal and neonatal characteristics. The third set of models were additionally adjusted for maternal history of risk behaviors and mental wellbeing. Apart from retaining all covariates, complications were mutually adjusted in the final model. All tests were two-tailed with *p* < 0.05 denoting statistical significance. Analyses were conducted using Statistical Package for Social Sciences (SPSS, version 25.0).

## 3. Results

This study recruited a total of 753 mothers (average age: 32.18 ± 4.51 years). Their average gestational age was 38.73 (SD = 1.44) weeks. Majority of them (89.8%) were married. There were 69.6% working full-time, 6.4% self-employed, 8.2% unemployed, and 15.1% as homemaker. More than 50% finished tertiary education or above. Regarding maternal history of risk behaviors, there were over 10% reported a history of tobacco use, 30% a history of alcohol drinking, and 0.3% a history of drug abuse. Their average monthly household income was the equivalent of USD 4,803 (SD = 3,095). The mean cord blood TL (*T*/*S* ratio) was 15.04 (SD = 6.25).

Based on hospital records from the CDARS, there were 232 mothers (30.8%) with infection, 226 (30.0%) with anaemia, and 45 (6.0%) with hypertensive disorder during pregnancy. Among mothers with infection, 160 (21.2%) were found to be group B streptococcus (GBS) carrier, and 96 (12.7%) had various infections including chorioamnionitis and vaginosis. Furthermore, 50.6% had unspecific complications at delivery, and 5.2% had neonates with low birthweight, and 12.1% had neonatal jaundice.  Table 1 shows the results of demographic comparisons between mothers in different pregnancy complication groups (no complication vs. having one complication vs. having two complications). Cord blood TL was also significantly shorter in mothers with two complications (*p* = 0.049). Additionally, mothers with any one of the complications during pregnancy were more likely to develop complications at delivery (*p* < 0.001).

Table 2 displays the results of TL comparisons between mothers with different demographics and complications during pregnancy. Analyses showed that the neonates of mothers with infection during pregnancy had significantly shorter TL (*p* = 0.043). In particular, neonates whose mothers were GBS carrier had shorter TL (*p* = 0.042). The cord blood TL were even shorter when the mothers had two complications during pregnancy (*p* = 0.005). Specifically, cord blood TL was the shortest when maternal infection during pregnancy and neonatal jaundice coexisted (*p* = 0.045). No significant differences in cord blood TL were found across other characteristics.

Table 3 shows the results of the regression analyses examining the independent and combined effects of the complications during pregnancy and at delivery on cord blood TL. The crude associations were first examined in Model 1. Our analyses showed that maternal infection during pregnancy was significantly associated with cord blood TL (*β* = −0.16, 95% confidence interval (CI) = −0.31 to −0.005, *p* = 0.042). Although there were no significant associations between cord blood TL and anaemia or hypertension (all *p* > 0.05), we found that having two pregnancy complications was also significantly associated with cord blood TL (*β* = −0.28, 95% CI = −0.53 to −0.04, *p* = 0.023). No significant associations with cord blood TL were observed in complications at delivery including low birth weight, neonatal jaundice, and unspecific complication (all *p* > 0.05). Furthermore, our regression analyses examining the interaction between maternal health complications and child complications at birth on cord blood TL found that the co-occurrence of maternal infection during pregnancy and delivery complications (*β* = −0.23, 95% CI = −0.41 to −0.06, *p* = 0.010) or maternal infection during pregnancy and neonatal jaundice (*β* = −0.43, 95% CI = −0.85 to −0.01, *p* = 0.045) was found to be associated with shorter cord blood TL. The significant associations remained unchanged in Model 2 after adjusting for confounders including mothers' age, education level, employment status, and neonatal gender and in Model 3 after further adjusting for maternal history of risk behaviors and mental health-related quality of life. After mutual adjustment in Model 4, maternal infection during pregnancy among all the obstetric and foetal outcomes was the strongest factor associated with cord blood TL (*β* = −0.18, 95% CI = −0.33 to −0.02, *p* = 0.026). Other maternal complications and complications at delivery were not significantly associated with cord blood TL (all *p* > 0.05).

## 4. Discussion

This study is among the first to examine the independent and combined effects of complications during pregnancy and at delivery on cord blood TL. Using the hospital record data, we found that maternal infection during pregnancy, predominantly by group B streptococcus, was the dominant factor affecting cord blood TL. Shorter TL was found in neonates of mothers with infection and those with both infection and anaemia, hypertension, delivery complications, or neonatal jaundice. Our results support the hypothesis that the quality of intrauterine environment is implicated in the dynamics of telomere after birth.

The detection of shorter TL in neonates born to mothers with pregnancy complication suggests that cord blood TL could be an important biomarker reflecting the impact of adverse intrauterine exposures at the cellular level. Previous studies have shown that telomere is the most vulnerable during the intrauterine period [20, 21]. In addition to genetic inheritance, it is well recognized that the intrauterine period is considered the critical period for foetal growth with rapid cell replication and differentiation. Foetal development has been described as a context-specific process wherein exposure to suboptimal intrauterine environment would induce structural and functional alternations in the developing foetuses [3, 31]. Our results further indicate the possible existence of interindividual variation in TL during the intrauterine period. Although the dynamics of TL at birth through early adulthood remains to be elucidated, previous studies have shown that the rate of telomere shortening reaches its peak in the first 4 years of childhood [32]. Different postulations were made over TL dynamics after early childhood. Some studies postulated that TL would reach a plateau and remain steady after early childhood [33], while others suggested a gradual reduction of TL throughout life [21]. To unravel the complexity in telomere dynamics, more longitudinal investigations should track TL and its associated factors from birth to adulthood.

In this study, maternal infection was the major pregnancy complication affecting the cord blood TL. Maternal viral or bacterial infections are common during pregnancy, and there is evidence that these infections can elevate the level of oxidative stress and trigger inflammatory responses in mothers [34, 35]. It is now widely considered that maternal infection is potentially harmful to the health and development of foetuses. Previous animal studies have demonstrated that maternal infection can evoke foetal systemic inflammatory responses indicated by the elevation of proinflammatory cytokine in the foetus or foetal environment [36]. Its potential underlying mechanisms include (i) the transplacental passage of maternal proinflammatory cytokines and reactive oxidative species, (ii) activation of cytokine production in the placenta by maternal derived cytokines, and (iii) transport of bacteria or cell wall lipopolysaccharide across the placenta [34, 36]. These responses together may impose oxidative stress on, and expose greater inflammatory load to, the developing foetus.

Telomere is particularly vulnerable to oxidative stress due to its rich content of guanine residues which can be easily oxidized to form oxidative lesion [27, 37]. When telomeric DNA becomes deficient in repairing single-strand breaks, its length can shorten at a faster pace [38]. Increased inflammatory load may also accelerate telomere shortening by promoting cell turnover and replicative senescence [37]. The combined effects of increased oxidative stress and inflammatory load may result in foetoplacental telomeric DNA damage which is a potential cause for accelerated telomere shortening [38]. Notably, neonates in this study were found to experience higher burden as reflected by shorter TL when their mother had infection plus anaemia or hypertension during pregnancy, delivery complications, or neonatal jaundice. However, in the absence of maternal infection during pregnancy, these complications had no effects on cord blood TL, perhaps because maternal infection is an important building block of the adverse intrauterine environment which may make the neonate's telomeres more susceptible to other complications. Prevention and early detection of maternal infection are recommended for the long-term health and wellbeing of mothers and their children.

To further elucidate the mechanism underlying the effect of maternal infection on cord blood TL, we extracted diagnosis details from the mothers' medical record and found that two-thirds of them were GBS carriers. The relatively high proportion of GBS in our study can be due to the universal GBS screening for pregnant women at Kwong Wah Hospital. GBS is an encapsulated Gram-positive bacterium which colonizes the lower gastrointestinal tract and the urogenital tract of 20–30% of healthy women [39]. Although most GBS infection might be asymptomatic, it is reported that GBS infection might activate the procell death pathways that could impose serious impacts on the health of both the foetus and the mothers during pregnancy. Previous studies on the host immune response to asymptomatic GBS colonization found elevated levels of immunoglobulins (IgA and IgG) [40], suggesting that asymptomatic GBS colonization may induce a localized or even systemic immune response in the pregnant women and in turn evoke systemic inflammatory responses in the foetus. Maternal GBS colonization was also suggested to be one major risk factor for neonatal disease. Neonates exposed to GBS from their mother are associated with increased risk for the development of early-onset disease including pneumonia and sepsis during the first few months of age [41]. Shorter cord blood TL detected in this group of exposed neonates provides further evidence on the negative health impacts of GBS infection to the neonates. Therefore, further evaluation on the necessity of early detection and treatment of asymptomatic GBS infection in pregnancy is indicated.

This study has several strengths. First, the hospital data was retrieved from the official electronic medical record system containing all outpatient attendances and inpatient admissions in local public hospitals. Hence, this study was able to provide accurate information on the patterns of morbidity and was able to minimize recall bias by not relying on self-report medical history. Moreover, this study collected cord blood to measure TL of the neonates which can give a reliable estimate of its linkages with antenatal factors. However, there are several limitations. First, subjects were recruited from a single antenatal clinic at a local public hospital. This might reduce the generalizability of our findings to the wider population groups. Second, we did not examine maternal or paternal TLs which are known to be associated with cord blood TL [9]. Examining the association of TL in parents and their neonates would permit the segregation of inheritance or genetics from other environmental factors and therefore advance our understanding of factors associated with the rate of telomere shortening during the intrauterine period.

In conclusion, this study demonstrated that after controlling for confounding factors, maternal infection during pregnancy was significantly associated with shorter cord blood TL in a unique manner such that its presence may critically determine the susceptibility of telomere to other factors. Namely, the presence of maternal infection during pregnancy exposes the TL of the neonates to the negative influence of maternal anaemia, hypertension, delivery complications, or neonatal jaundice. Our findings have thus uncovered a form of interaction between known intrauterine adversities on TL reduction hitherto not specified in the literature. Given that shorter TL at birth is implicated in the development of health risks and problems later in life, it is imperative to conduct longitudinal studies to investigate whether differences in TL at birth would increase the susceptibility to disease in childhood and later periods.

## Figures and Tables

**Table 1:** Demographics and clinical diagnoses of study participants (*N* = 753).

		Number of complication during pregnancy			
	TOTAL (*N* = 753)	None (*N* = 331)	One (*N* = 343)	Two (*N* = 79)	
	*N* (%)/mean (SD)				*p*
Sex of the baby					0.202
Male	392 (52.1%)	162 (48.9%)	191 (55.7%)	39 (49.4%)	
Female	360 (47.8%)	168 (50.8%)	152 (44.3%)	40 (50.6%)	
Maternal age	32.18 (4.51)	32.34 (4.49)	31.98 (4.54)	32.58 (4.53)	0.434
Marital status					0.904
Single/divorced	58 (7.7%)	25 (7.6%)	26 (7.6%)	7 (8.9%)	
Married	676 (89.8%)	300 (90.6%)	307 (89.5%)	69 (87.3%)	
Maternal education level					0.262
Junior secondary school or below	89 (11.8%)	36 (10.9%)	44 (12.8%)	9 (11.4%)	
Senior secondary school	236 (31.3%)	97 (29.3%)	110 (32.1%)	29 (36.7%)	
Tertiary education or above	425 (56.4%)	198 (59.8%)	187 (54.5%)	40 (50.6%)	
Maternal employment status					0.091
Full-time	524 (69.6%)	225 (68.0%)	248 (72.3%)	51 (64.6%)	
Self-employed	48 (6.4%)	17 (5.1%)	27 (7.9%)	4 (5.1%)	
Homemaker	114 (15.1%)	61 (18.4%)	37 (10.8%)	16 (20.3%)	
Students	2 (0.3%)	2 (0.6%)	0 (0.0%)	0 (0.0%)	
Unemployed	62 (8.2%)	26 (7.9%)	29 (8.5%)	7 (8.9%)	
Family monthly income (USD)	4,803 (3,095)	4936 (3175)	4711 (3024)	4690 (3070)	0.618
Maternal SF12v2 mental component score	48.40 (8.70)	48.84 (7.88)	48.47 (9.03)	46.41 (10.2)	0.084
Neonatal telomere length (*T*/*S* ratio)	15.04 (6.25)	15.31 (6.61)	15.05 (6.2)	13.53 (4.45)	**0.049** ^∗^
Gestational age	38.73 (1.44)	38.67 (1.49)	38.78 (1.43)	38.75 (1.31)	0.735
Maternal history of risk behaviors					
Ever smoked	88 (11.7%)	38 (11.5%)	40 (11.7%)	10 (12.7%)	0.947
Ever consumed alcoholic drink	226 (30.0%)	89 (26.9%)	108 (31.5%)	29 (36.7%)	0.144
Ever engaged in drug abuse	2 (0.3%)	1 (0.3%)	1 (0.3%)	0 (0.0%)	0.890
Maternal health complication during pregnancy					
Maternal infection	232 (30.8%)	—	—	—	
Group B streptococcus carrier	160 (21.2%)	—	—	—	
Other types of infection (e.g. chorioamnionitis, vaginosis)	96 (12.7%)	—	—	—	
Anaemia	226 (30.0%)	—	—	—	
Hypertension	45 (6.0%)	—	—	—	
Total number of complications during delivery					
Having one of the above diagnoses	343 (45.6%)	—	—	—	
Having two of the above diagnoses	79 (10.5%)	—	—	—	
Infection & anaemia	59 (7.8%)	—	—	—	
Infection & hypertension	11 (1.5%)	—	—	—	
Anaemia & hypertension	9 (1.2%)	—	—	—	
Complication at delivery					
Unspecific complications	381 (50.6%)	58 (17.5%)	248 (72.3%)	75 (94.9%)	<0.001^∗∗∗^
Low birthweight	39 (5.2%)	20 (6.0%)	16 (4.7%)	3 (3.8%)	0.608
Neonatal jaundice	91 (12.1%)	47 (14.2%)	40 (11.7%)	4 (5.1%)	0.077
Total number of complications at delivery					<0.001^∗∗∗^
Having one of the above diagnoses	411 (54.6%)	105 (31.7%)	234 (68.2%)	72 (91.1%)	
Having two of the above diagnoses	50 (6.6%)	10 (3.0%)	35 (10.2%)	5 (6.3%)	

^∗^*p* < 0.05; ^∗∗∗^*p* < 0.001.

**Table 2:** Telomere length of mothers with different demographics and clinical diagnoses.

		Telomere length (*T*/*S* ratio)		Comparison^#^
		Mean (SD)		
	N (%)	Yes	No	*p* value
Demographics				
Baby sex—male	392 (52.1%)	14.65 (5.94)	15.41 (6.55)	0.100
Parents being single/divorced	58 (7.7%)	15.28 (4.44)	15.02 (6.42)	0.757
Mothers received education from junior secondary school or below	89 (11.8%)	15.45 (5.85)	14.99 (6.30)	0.508
Mothers being full-time worker	524 (69.6%)	15.09 (6.31)	14.94 (6.12)	0.762
Maternal history of risk behaviors				
Ever smoked	88 (11.7%)	15.28 (4.83)	15.01 (6.42)	0.701
Ever consumed alcoholic drink	226 (30.0%)	15.00 (5.53)	15.06 (6.54)	0.902
Ever engaged in drug abuse	2 (0.3%)	15.98 (4.66)	15.04 (6.25)	0.831
Clinical diagnoses				
*Maternal health complication during pregnancy*				
Any type of maternal infection	231 (30.7%)	14.31 (5.14)	15.32 (6.69)	0.043^∗^
Group B streptococcus carrier	160 (21.2%)	14.11 (4.62)	15.25 (6.60)	0.042^∗^
Other types of infection (e.g., chorioamnionitis, vaginosis)	96 (12.7%)	14.38 (5.62)	15.10 (6.33)	0.292
Anaemia	226 (30.0%)	14.93 (6.11)	15.04 (6.31)	0.829
Hypertension	45 (6.0%)	14.14 (6.68)	15.06 (6.22)	0.345
Any one of the above diagnoses	342 (45.4%)	15.05 (6.20)	15.31 (6.61)	0.638
Any two of the above diagnoses	78 (10.4%)	13.53 (4.45)	15.31 (6.61)	0.005^∗∗^
*Complication at or after delivery*				
Unspecific complications	366 (48.6%)	14.78 (5.81)	15.25 (6.66)	0.288
Low birthweight	39 (5.2%)	15.16 (5.09)	15.00 (6.31)	0.869
Neonatal jaundice	91 (12.1%)	14.31 (5.02)	15.04 (6.30)	0.419
Any one of the above diagnoses	411 (54.6%)	14.82 (6.00)	15.36 (6.69)	0.265
Any two of the above diagnoses	50 (6.6%)	14.47 (5.48)	15.36 (6.69)	0.375
*Maternal* *health* *complication* × *child* *complication*				
Maternal infection x unspecific complications at delivery	148 (19.7%)	13.83 (4.54)	15.30 (6.59)	0.002^∗∗^
Maternal infection × low birthweight	13 (1.7%)	13.94 (2.86)	15.03 (6.29)	0.534
Maternal infection × neonatal jaundice	21 (2.8%)	12.39 (6.71)	15.08 (6.22)	0.045^∗^

^#^Comparison was conducted using independent *T*-test; ^∗^*p* < 0.05; ^∗∗^*p* < 0.01.

**Table 3:** Associations between clinical diagnoses during pregnancy and neonatal telomere length.

	Model 1: crude associations		Model 2: further adjusted for maternal demographics and newborn characteristics^+^		Model 3: further adjusted for maternal risk behaviors and mental component of quality of life^#^		Model 4: further adjusted for each other^$^	
	*β* (95% CI)	*p*	*β* (95% CI)	*p*	*β* (95% CI)	*p*	*β* (95% CI)	*p*
Maternal health complication during pregnancy								
Maternal infection	-0.16 (-0.31, -0.005)	0.042	-0.17 (-0.33, -0.02)	0.027	-0.17 (-0.33, -0.02)	0.028	-0.18 (-0.33, -0.02)	0.026
Anaemia	-0.02 (-0.17, 0.14)	0.831	0.00 (-0.16, 0.15)	0.987	0.00 (-0.16, 0.16)	0.999	-0.01 (-0.17, 0.14)	0.870
Hypertension	-0.15 (-0.45, 0.16)	0.344	-0.16 (-0.47, 0.14)	0.287	-0.16 (-0.47, 0.14)	0.294	-0.17 (-0.47, 0.13)	0.269
Any two of the above diagnoses	-0.28 (-0.53, -0.04)	0.023	-0.29 (-0.53, -0.05)	0.020	-0.29 (-0.53, -0.04)	0.021	—	—
Complication at delivery								
Unspecific complications	-0.08 (-0.22, 0.07)	0.287	-0.08 (-0.22, 0.06)	0.258	-0.08 (-0.23, 0.06)	0.260	-0.09 (-0.23, 0.06)	0.232
Low birthweight	0.03 (-0.29, 0.35)	0.869	0.03 (-0.29, 0.35)	0.842	0.03 (-0.29, 0.36)	0.834	0.00 (-0.32, 0.33)	0.976
Neonatal jaundice	-0.12 (-0.33, 0.10)	0.294	-0.10 (-0.32, 0.12)	0.366	-0.10 (-0.32, 0.12)	0.369	-0.11 (-0.33, 0.11)	0.325
Any two of the above diagnoses	-0.14 (-0.44, 0.16)	0.352	-0.15 (-0.45, 0.15)	0.317	-0.15 (-0.45, 0.14)	0.314	—	—
Maternal health complication × child complication								
Maternal infection x unspecific complications at delivery	-0.23 (-0.41, -0.06)	0.010	-0.25 (-0.42, -0.07)	0.006	-0.25 (-0.42, -0.07)	0.007	—	—
Maternal infection × low birthweight	-0.17 (-0.72, 0.37)	0.533	-0.17 (-0.71, 0.38)	0.543	-0.16 (-0.71, 0.38)	0.556	—	—
Maternal infection × neonatal jaundice	-0.43 (-0.85, -0.01)	0.045	-0.45 (-0.88, -0.02)	0.040	-0.45 (-0.88, -0.02)	0.040	—	—

^+^Further adjusted for mother's age, educational level, employment status, and infant's sex; ^#^further adjusted for history of smoking, being drunk, drug usage of the mothers, and the mothers' mental component of quality of life; ^$^further adjusted for each other's health complications.

## Data Availability

The data that support the findings of this study are available from the corresponding author, Dr. Patrick Ip, upon reasonable request.

## Abbreviations

TL: Telomere length.
